# Why young people stop taking their attention deficit hyperactivity disorder medication: A thematic analysis of interviews with young people

**DOI:** 10.1111/cch.12978

**Published:** 2022-02-20

**Authors:** Daniel Titheradge, Jo Godfrey, Helen Eke, Anna Price, Tamsin Ford, Astrid Janssens

**Affiliations:** ^1^ College of Medicine and Health University of Exeter Exeter UK; ^2^ Population Health Sciences, School of Physiology, Pharmacology and Neuroscience University of Bristol Bristol UK; ^3^ Livewell Southwest Plymouth UK; ^4^ Department of Psychiatry University of Cambridge Cambridge UK; ^5^ Department of Public Health University of Southern Denmark Odense Denmark

**Keywords:** adolescence, attention deficit hyperactivity disorder (ADHD), CATCh‐uS, medication, transition

## Abstract

**Background:**

Attention deficit hyperactivity disorder (ADHD) is a common neurodevelopmental disorder that can persist into adulthood. Young people often stop taking ADHD medication during adolescence despite evidence that continuation would be beneficial. Increasingly, young people are restarting medication in early adulthood suggesting that cessation was premature. In this paper we explore the reasons given by young people for discontinuing ADHD medication.

**Methods:**

Qualitative data from the Children and Adolescents with ADHD in Transition between Children's and Adult Services (CATCh‐uS) project was analysed to look for reasons for stopping medication. Semi‐structured interviews with three groups of young people were analysed using thematic and framework analysis; this included young people prior to transition (*n* = 21); young people that had successfully transitioned to adult services (*n* = 22); and young people who left children's services prior to transition but re‐entered adult services later (*n* = 21).

**Results:**

Reasons given by young people for stopping ADHD medication included the following: the perceived balance between benefits and adverse effects of medication; perceptions of ADHD as a childhood or educational disorder; life circumstance of the young person and challenges young people faced in accessing services.

**Conclusions:**

A multidimensional approach is needed to address discontinuation of ADHD medication in order to improve the long‐term prospects and quality of life for these young people. Possible approaches include access to non‐pharmacological treatments and improved psychoeducation. As many reasons given by young people are not unique to ADHD, these findings are also of relevance to medication adherence in other chronic childhood conditions.

**Key Messages**

- Continuing ADHD medication into adulthood results in improved long‐term health, social and occupational outcomes, however, young people frequently stop their medication despite ongoing symptoms.
- This qualitative study explores the reasons that young people give for stopping ADHD medication, and consideration of the themes identified will enable practicing clinicians to enhance adherence to treatment with the potential for improved outcomes.
- The reasons given by young people for stopping medication are not unique to ADHD and have wider relevance to medication adherence in other long‐term conditions.

## 1 INTRODUCTION

### 1.1 Background

Attention deficit hyperactivity disorder (ADHD) is a neurodevelopmental condition estimated to have a community prevalence in children of 5% globally (Sayal et al., 2018). It is characterized by difficulties in attention, hyperactivity and impulsivity that are present in at least two settings and are inappropriate for the developmental stage of the child. ADHD can be treated through the use of medication including methylphenidate, lisdexamfetamine, dexamfetamine, atomoxetine or guanfacine.(National Institute for Health and Care Excellence (NICE), 2018) Historically, ADHD was understood to be a childhood disorder, but is increasingly recognized as a chronic condition which can persist into adulthood (Sibley et al., 2016). Young people with ADHD are more likely to experience poorer health, social, educational and occupational outcomes compared to their peers without ADHD (Montano & Young, 2012). Treatment of ADHD with medication in children, adolescents and adults is effective in reducing symptoms, reducing functional impairment and improving quality of life (Cortese, 2020). In addition, ADHD medication is associated with a number of long‐term benefits including improved academic performance, improved occupational function, reduced rates of substance abuse and reduced antisocial behaviour (Shaw et al., 2012). Prescription of ADHD medication in the United Kingdom (UK) declines more steeply than epidemiological estimates would predict between the ages of 16 and 18 given the estimated rate of symptom persistence (Newlove‐Delgado et al., 2019; Wong et al., 2009). The median time to stopping medication from the age of 16 was 1.51 years for young people with ADHD in the UK (Newlove‐Delgado et al., 2019), which is in line with recent data for the UK showing that less than one out of five young people with ADHD in need of transition access adult mental health services (Eke et al., 2019).

### 1.2 Context

Understanding the reasons for discontinuation of ADHD medication from the perspective of young people is important. Evidence from a systematic review suggests that the reasons given for discontinuation in research studies involving young people differs from published expert opinion and the authors highlight the need for further research into the reasons for discontinuation of medication, which we seek to address in this paper (Gajria et al., 2014). Analysis of research studies involving young people from this systematic review identified adverse effects and ineffectiveness of medication as the leading causes for discontinuation (Gajria et al., 2014). The authors conducted the same analysis on expert opinion articles written by clinicians and researchers and found that adverse effects of medication were still cited as the leading cause for discontinuation of ADHD medication, however, expert opinion articles appear to overlook the importance of medication ineffectiveness, and cited other causes of medication discontinuation (e.g., dosing inconvenience, patient attitude and social stigma) more frequently than the young people in the primary research studies (Gajria et al., 2014). Research studies on medication discontinuation in young people use a variety of approaches to identify the reason for medication discontinuation, including retrospective review of records, parent/caregiver perspectives and clinician perspectives, all of which may fail to capture the voice of the young person.

Although healthcare transition issues have been studied for young people with ADHD (Price, Janssens, et al., 2019), few studies have explored the reasons underlying the discontinuation of ADHD medication from the perspective of young people. A qualitative study of seven young people at transition age in UK highlighted that young people thought that they took medication ‘for school’ (Newlove‐Delgado et al., 2018). A small number of studies from the United States (US) offer further insights. A study of 44 young people aged 13 to 18 years old, identified that adverse effects were a key contributor to negative feelings about medication and a major reason to stop taking it; some young people in this study also questioned the utility of ADHD medication once school was finished (Brinkman et al., 2012). Ten young people from the US, who had recently started college (higher education), reported their challenges of medication adherence during this transition. Their reasons for stopping ADHD medication included that some simply did not like taking it, whilst others were unable to manage their medication independently (Schaefer et al., 2017). Data from parents of thirteen young people starting college in the US identified the impact of medication on personality and ability to make friendships; career goals including joining the military; adverse effects and medication‐related beliefs (including the belief that medication was no longer required in early adulthood) on adherence to ADHD medication (Schaefer et al., 2018). A study of parents' and young people's perspectives on ADHD treatment suggested consistency between child and parent attitudes towards ADHD treatment (Berger et al., 2008). A recent review of medication adherence in children, adolescents, and adults with ADHD, identified factors including fear of addiction; medication effectiveness; psychiatric comorbidity and medication side effects to influence adherence (Khan & Aslani, 2019).

The current paper describes the reasons given by a larger sample of young people in the UK for stopping medication for ADHD. The young people shared their experiences of being prescribed ADHD medication during interviews conducted as part of the Children and Adolescents with ADHD in Transition between Children's and Adult Services (CATCh‐uS) study; a study on the transition of young people with ADHD from children's to adult services (Janssens et al., n.d.).

## 2 METHODS

**FIGURE 1:**
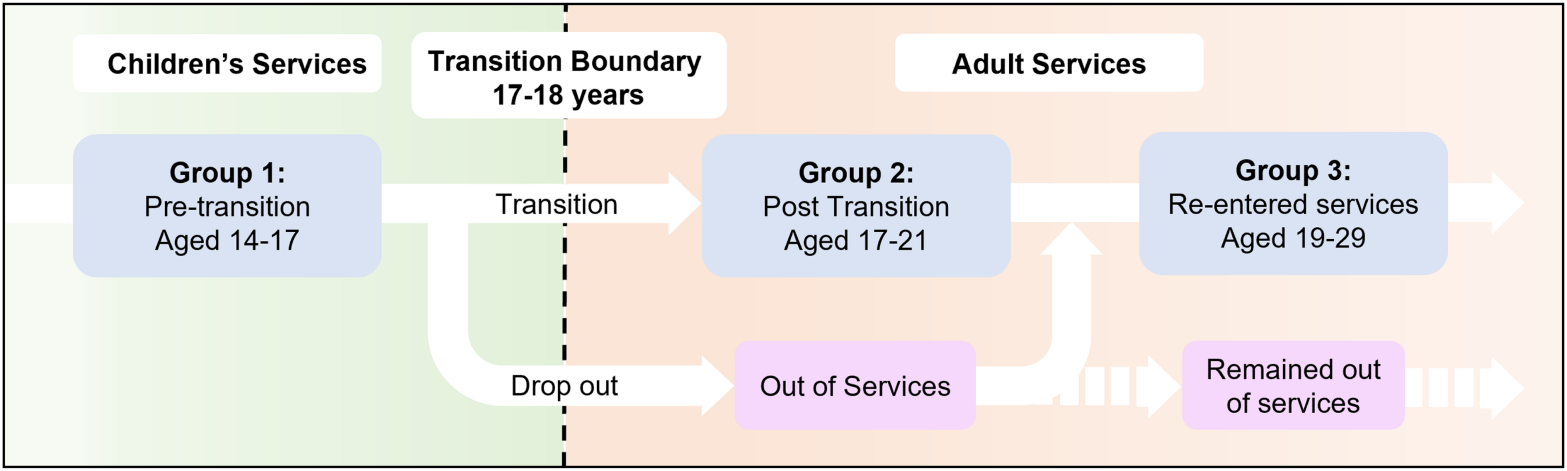
Diagram illustrating the three groups of young people recruited to Children's and Adult Services (CATCh‐uS)

CATCh‐uS interviewed three groups of young people with ADHD at different stages of the transition process. These were pre‐transition (*n* = 21); at completion of the transition process (*n* = 22; referral accepted and or attended first appointment in adult services); and after a failed or non‐existent transition and successful re‐entry into adult services after a gap in service access of at least one year (*n* = 21; see Figure 1). For full details of the recruitment strategy please see the CATCh‐uS report (Janssens et al., n.d.).

Young people were recruited from ten health services that provided ADHD treatments for the National Health Service (NHS Trusts). Five NHS trusts were purposefully selected to represent geographical spread and a range of service models for the provision of ADHD care (South London and Maudsley NHS Foundation Trust, Berkshire Healthcare Foundation NHS Trust, Devon Partnership NHS Trust, Coventry & Warwickshire Partnership Trust, Nottinghamshire Healthcare NHS Foundation Trust). Five further NHS Trusts volunteered to support the recruitment after the study had started via the National Institute for Health Research (NIHR) Clinical Research Network (South Staffordshire & Shropshire Foundation Trust, Leicestershire Partnership NHS Trust, Lincolnshire Partnership NHS Foundation Trust, Somerset Partnership NHS Foundation Trust and Sussex Community NHS Foundation Trust).

Interviews were conducted at the convenience of the young person and a place of their choosing, which included at home, a public place, or the clinic where the young person received treatment. Decisions about sample size drew on experiences of previous studies on transition (Beresford & Stuttard, 2014; Mitchell & Beresford, 2014). We applied an iterative approach to data collection and analysis, using the analytic process of constant comparison to monitor data quality and value, and the sampling framework and size was adjusted accordingly to ensure as broad a representation of experience as possible. As advised by the NHS Research Ethics Committee (REC), we stopped recruitment once data saturation was reached to avoid unnecessary interviews.

Three researchers (HE, AP and AJ) conducted semi‐structured interviews using a topic guide informed by existing literature on transition and input from the project's parent advisory group, which covered the following topics:

- current and future medication use,
- current and future contact with services,
- preparation for and/or experiences of the transition process,
- views on key elements of optimal transition.

All interviews were digitally voice‐recorded and transcribed verbatim. NVivo 11 (QSR International, Brisbane) was used to manage the data and the analysis. Data analysis followed a Framework Analysis approach, which is an approach to thematic analysis that has been explicitly developed in the context of applied social science research (Gale et al., 2013). Data for each of the groups were analysed separately and then compared. After a detailed examination of the transcripts, a team of four (AJ, AP, HE and AS) indexed three to four interviews. Indexing was discussed by the group to identify and label codes. These codes were used to create a coding tree that was applied to the remaining interviews. The final stage involved creating a framework matrix; interviews represented rows and codes columns. The framework approach allowed themes within a framework and between the three frameworks, to be compared and contrasted, to identify patterns or links, and to provide explanations of the findings (Janssens et al., n.d.). For the current paper, themes related to medication were extracted from each of the young people framework summary matrices and analysed by two researchers (DT and JG). All identified reasons for stopping medication, within each of the groups of young people, were then synthesized across the three groups.

Ethical approval for the study was granted by NRES South Yorkshire Ethics Committee: Yorkshire & The Humber (REC Reference: 15/YH/0426) and the University of Exeter Medical School ethics board (REC Application Number: 15/07/070). Written informed consent was obtained from all participants prior to the interview taking place. Parents of young people aged 14 to 16 years old consented for their child to take part; researchers sought assent from the young person. A £10 shopping voucher was offered to thank young people and support their recruitment.

## 3 RESULTS

A total of 64 young people at three different stages of, transition, were recruited and interviewed from 10 NHS Trusts across England (Table 1) (Janssens et al., n.d.). All young people interviewed were attending NHS services for their ADHD, and most reported being on ADHD medication, at the time of the interview.

**TABLE 1:** Stage, gender and age‐range of participants

Stage	Gender		Age range	Total
	M	F		
**Pre transition**	16	5	14–17	21
**At transition**	13	9	17–21	22
**No transition (re‐entered as adult)**	15	6	19–29	21
**Total**	44	20	14–29	64

*Note*: M = Male, F=Female.

The reasons given by young people for stopping medication for ADHD were grouped into four key themes: the perceived balance between benefits and adverse effects of medication; perceptions of ADHD as a childhood or educational disorder; the life circumstances of the young person; and the challenges young people faced in accessing services. The findings from these themes are described with quotes illustrating each theme and subtheme in Table 2.

**TABLE 2:** Quotes to illustrate themes identified in the study

	Subtheme	Quote
**Balance between benefits and adverse effects of medication**	Weighing adverse effects and benefits	*‘They were turning out to be more bad than good’*
		*‘So the medication, it helps but it's the side effects of the medication that I didn't really like. That's why I was very rebellious against taking the medication at some points’*
	Benefits	*‘The first day I went into school after taking the medication and I remember we had a test and my teacher got the results from this test, I think it was in maths, and they were, like, “Is this yours?” It was perfect. Every answer was right’*
		*The medication helped me to concentrate and keep me in control when I was in school. Since then the medication has been really good, it works really well, especially when I'm in college and at work*.
		‘*I need this medication, I want this medication and I need it to survive in this world, basically, and if I don't take this medication this world won't accept me’*
	Ineffectiveness	*‘But I knew straightaway that when I got given that Ritalin that it was shocking. It didn't work at all’*
		*‘it will all start to become ineffective slowly but surely’*
	Dependence	*‘I don't want to get dependent by them, after a time I think I should just stop and maybe even move on to something else. I've got to keep on moving medication because it's not good for just staying on one lot of medication because it could damage you’*
	Side Effects	*‘I was waking up every morning being sick, without fail, every morning. I was pale. I was white as a ghost. I wasn't eating, I weren't sleeping, I weren't drinking’*
	Medication trials	*‘At first I was fine with it, but I've been changed to like five different ones. So I think it's sort of boring trying, because it's different side effects on all different ones. But it does the job when I'm at college, so’*.
	Effects on identity	*‘… it makes you different, and act differently, and your characteristics and all that, then you feel like you're not yourself. And actually, if you're not being yourself then it can affect your identity’*
	Comorbidity	*‘… I also have anxiety as well, so there's always a big balance between medication for this and medication for that … I can concentrate so well now, I'm also concentrating on the bad bits, the anxiety’*
	Feeling different	*‘I feel like ‘oh why should I have to take that?’ I just try and feel normal’*
**Perceptions of ADHD**	ADHD will get better	*‘As soon as I found out I had it, every single person told me, even medical professionals, they all told me, “It will go away when you're about 17 or 18 and you'll be a normal person”’*
		*‘… it will probably calm down a bit more when I get older. But I will still always be that hyperactive child, and that's the ADHD in me’*
	Medication is to cope in school	*‘I think they targeted school as that was the battlefield that I needed to get beyond to give me a step in later life and once that was achieved it was, yes, you can do it on your own, you don't need us anymore*’.
		*‘To be honest, I kind of felt like the school were just happy I'd take the medication because then I'd be like, not out the way but I'd just be quiet and just shut up, not really say anything’*.
		*‘… I was just finishing school at the time and for a couple of years I didn't do anything. I wasn't at school so I didn't take the medication. I was only initially taking it for school’*.
		*‘I finished school and I went to work, I didn't think I needed it anymore and I quit’*.
	Perceived ability to cope	*‘I did the stupid thing that's all teenagers who know better do, especially when they're on medication and they see that they are coping and don't actually tie it back to the fact that it's the medication that's meaning that they can cope. I took myself off the medication. I lasted a good couple of years before anything went sideways but then it went sideways in a big way’*.
		*‘I don't know why I stopped engaging because I was very in denial at one point. I was like, ‘I don't need that. It's fine. I can manage myself,’ but it turns out that I really can't’*.
	Relationship to higher education	*‘I think definitely in the first year of university I'll need it because I find, especially with the anxiety and the ADHD, they kind of flare up when I'm put in a new environment’*
**Life circumstances of the young person**	Moving	*‘So I was up in [another county], I then moved back down and basically I got lost out of the system’*.
		*‘I moved a little while ago, and they said that they tried to get in contact with me, and I missed an appointment. And because I missed one appointment they just dropped me off the services’*
		*‘Young Person: Yes. My first year at college was a flop anyway. I moved in with my dad, who doesn't work … He didn't have the support that I needed to push me. So I ended up missing lectures if that's what you call them at college. Lessons, whatever, and then didn't turn up. Wasn't taking my medication and stuff like that and my education just stopped’*.
	Chaotic Lifestyle	*‘Well basically I dropped out … It's not like I dropped out, it's because I was obviously getting to that age where I wasn't in there no more, so they obviously dropped me out. I was going through hostels. Like I said, it was a bit of a weird situation where I didn't know what was actually going off’*
	Planning pregnancy	*‘Well they said that I would have to have contraception or the tablets but can't have the tablets and get pregnant’*.
	Prison	*‘I forgot to take my tablet one day and I ended up getting sent down [to prison] … When I came out of prison my behaviour was getting even more worse and it was coming to the point where I was getting stopped every single day by police because of my behaviour. I thought well I'm on licence, I'm going to come back to my tablets and get more help because obviously I've got a baby on the way now and I need my tablets’*.
	Continuing medication depends on future job	*‘I don't know how long I'm going to stay on it because, as you mentioned earlier, the whole idea going into the army and military, they don't really want you on medication because that's a bit of a liability’*
		*Interviewer: As you're getting older is there any reason why you would still continue it?*
		*‘Young person: If I get a job that I don't like or something I probably would’*.
	Concerns about driving on medication	*‘There's one thing that we've come up against lately with my medication is because I was wanting to start driving but because it's a controlled drug – [parent overspeaking] – I could get done for drug driving, it's against the law, unless I get a certificate from the doctor saying I'm allowed to drive. But no doctor has given a certificate out’*
**Challenges in accessing services**	Difficulties in accessing appointments and prescriptions	*‘Interviewer: So is there anything that worries you about moving into an adult service?’*
		*‘Young Person: Not getting my tablets anymore’*.
		*‘Interviewer: Is there anything else that you think might make you stop taking the medication?’*
		*‘Young person: Personally, no, as long as I've got access to it with a local GP wherever I am then I'd be happy to continue with it really, unless there was some kind of symptoms that it could bring up, then I might feel inclined to stop’*.
		*‘It is a lot of dedication and a lot of money that I have to spend to get. So about £20 every time I go and see them. And another £8 to get the prescription’*.
		*‘I was in college. I had just started college and I thought I was doing really well, I was like I'm doing really well, I don't need it, I hate the constant appointments, because I used to have to travel to [Town] for my appointments’*.

### 3.1 Theme 1: The perceived balance between benefits and adverse effects of medication

The perceived balance between benefits and adverse effects of medication covered several issues: the (in)effectivity of the medication (*it works*), the physical and social changes caused by the medication, interaction with comorbid conditions, concerns about dependency and loss of normality due to the need to take regular medication. Most young people made decisions on whether to take medication based on the balance of their perception of the positive and negative impacts of the medication. Not surprisingly, young people tended to stop medication when the adverse effects were perceived to outweigh the benefits.

Young people had a variety of experiences with ADHD medication regarding effectiveness. Some mentioned that the medication was instantly beneficial with positive effects that included being more able to focus on work, manage their behaviour in school, stay still, avoid distraction, stabilize their mood, reduce anger and feel calmer. One participant explained that the medication *worked* and was their ticket to normality; they felt strongly about continuing their ADHD medication in order to fit in and conform whilst others questioned effectiveness from the outset. Some young people recognized that whilst they did not notice a difference, their parents or teachers had identified positive changes in their behaviour after starting medication. Some young people assumed that medication would gradually become less effective over time. A young person pre‐transition stated that *‘it will all start to become ineffective slowly but surely’*, and they expressed concern that if their medication were no longer effective this would have a negative impact on their future. Some young people had personally experienced this, whilst others were concerned about becoming reliant or dependent on medication and were concerned about long‐term effects.

Negative effects were discussed by young people in all groups; they reported similar adverse effects from their ADHD medication, including: anger, anxiety, appetite suppression, cardiac problems, dizziness, dry mouth, fainting, fatigue, gastrointestinal complications, hair loss, headache, insomnia, loss of motivation, low mood, overstimulation, seizures and weight loss. Although they were commonly reported, not all young people reported adverse effects and those reported ranged from mild to severe. Sometimes medication was changed, or the dose reduced by clinicians to deal with adverse effects, and sometimes medication was stopped altogether by clinicians, parents, or young people because adverse effects were intolerable.

Young people reported changes in thinking and behaviour, which were also cited as reasons to stop the medication. Some young people expressed that the medication made them feel vulnerable whilst others expressed that they would be *stronger* if they were not on medication. Some young people had concerns about, or stopped taking, medication as pre‐existing anxiety or depression was aggravated by ADHD medication. Young people also described feeling different from peers due to both their diagnosis of ADHD and the practicalities of taking medication. Taking medication made them *different* from the others, whilst also being a constant reminder of their differentness. For some young people this led to a desire to stop medication in order to feel *normal*.

### 3.2 Theme 2: Perceptions of ADHD as a childhood or educational disorder

Most young people viewed ADHD as a disorder that would no longer require treatment as they got older. We identified two pathways to this ‘conclusion’. First, the understanding that ADHD is a disorder of childhood and that they would grow out of it; an assumption that had its roots in their understanding of ADHD as it was explained to them. Young people reported clinicians advising them that ADHD would get better in the future, suggesting that some clinicians still view ADHD as a disorder of childhood. Second, the young people in this study were all diagnosed whilst in primary or secondary education and frequently identified their ADHD treatment as a way of coping in school; many believed that medication was no longer required once they finished education. As a result of the association between ADHD and school, many young people experimented with being off medication during weekends and holidays and reported that they needed the medication most when they had to *concentrate* at school.

Due to a lack of knowledge of what ADHD is and its prognosis, some young people interpreted improvements or changes in their symptoms as indicating that they no longer had ADHD. Those who re‐entered services in adulthood reflected that life had become more difficult after stopping medication. Through experience these young people identified that ADHD was not just a childhood disorder, and that they benefitted from continued medication and contact with services as they got older. Most who continued on to higher education chose to continue their medication, although some perceived education transition points as an opportunity to try to cope without medication.

### 3.3 Theme 3: Life circumstance of the young person

Changes in life circumstances were a common reason for young people to stop medication, although not always intentionally. These included: moving to a new area, moving to live with a different parent, pregnancy, future career intentions, and being sent to prison. Common explanations for unintentional medication cessation included being lost to clinical follow‐up, and a loss of parental support.

There were young people in both post‐transition groups who stopped medication because they were pregnant or were planning to become pregnant. One young person reported that they stopped going to children's services after being sent to prison and subsequently re‐entered ADHD services in adulthood after a period without medication. Several young people acknowledged that continuing ADHD medication would be dependent on their future job and what it entailed. Some young people suggested that their wish to learn to drive may be a reason that they would consider stopping their medication, as they believed it would be prohibited.

### 3.4 Theme 4: Challenges young people faced in accessing services

Challenges in accessing services were mentioned by young people in all three groups and directly led to medication discontinuation for some young people. Difficulties were most commonly reported in relation to the transition from children's to adult services. Reported problems included discharge from services due to perceived non‐engagement, a lack of onward referral, prescription costs and the length and cost of travel to appointments. One young person acknowledged the importance of having access to a local GP in order to be able to continue taking medication.

The group that re‐entered adult services gave the following additional service‐related reasons for having stopped their medication; their prescribing doctor had retired, or they were either not referred on to adult services, or the referral was not accepted. Practical difficulties of engaging with adult services were mentioned repeatedly, such as lack of parental support to remind them, take them or pay for travel to appointments as well as helping/reminding them to pick up prescriptions.

## 4 DISCUSSION

In this study we explored the reasons given by young people for stopping ADHD medication, which revealed four broad themes; the perceived balance between benefits and adverse effects of medication; perceptions of ADHD; life circumstances; and challenges faced accessing services. Whilst their stories showed some overlap and could be organized into the themes presented, ultimately each young person described a unique combination of reasons and input that led to their decision to stop medication. Whilst the identified themes are distinct, they also impact upon and interact with one another; for example, when considering the balance between benefits and adverse effects of medication, a young person's beliefs about the prognosis of ADHD will be relevant in reaching the decision to stop medication.

### 4.1 The perceived balance between benefits and adverse effects of medication

The issues described in this theme indicate that taking ADHD medication has a significant and varied impact on young people. Our finding that adverse effects are a reason to stop medication is supported by several studies with adolescents and college students (Brinkman et al., 2012; Schaefer et al., 2017; Wong et al., 2009). Inadequate optimization of medication may contribute to beliefs about medication ineffectiveness (Adamou & Bowers, 2011), and young people may be more likely to stop treatment as a result of the tipping balance between adverse effects and benefits. A recent systematic review reported that effectiveness and adverse effects influenced initiation, implementation and discontinuation of ADHD medication (Khan & Aslani, 2019). Professionals must ensure that treatment is optimized to ensure maximum benefit in the hope that young people will value and continue medication when it is indicated.

The health belief model, as described by Becker et al., (Becker & Maiman, 1975) posits that a variety of key beliefs (likelihood of a health problem, severity of the consequences, benefits and costs of a preventive behaviour) shape health‐related behaviour (Abraham & Sheeran, 2015). This model does not fully explain why the young people in this study decided to stop their medication. An alternative understanding of healthcare behaviour is proposed by Conrad (1985) who suggests that decisions about medication are also influenced by the ability to self‐regulate illness, achieving normality, and the impact of stigma (Conrad, 1985). Our findings also support the Conrad model, with young people discussing the concepts of differentness and normality. Neither model, however, covers the wide variety and complexity of the interaction between the themes identified in this study.

### 4.2 Perceptions of ADHD as a childhood or educational disorder

Our data show that medication discontinuation is frequently related to a lack of understanding of the possible lifelong nature and impact of ADHD, which is reinforced by services discharging young people at the age of 18; these findings were echoed by parallel studies based on the CATCh‐uS project (Price, Newlove‐Delgado, et al., 2019). Despite increasing evidence that ADHD is a long‐term condition for many diagnosed in childhood (Sibley et al., 2016), our data suggest that this is not the perspective held by many young people or their families. Indeed, in other research the question most young people wanted to ask their clinician was ‘Will I grow out of ADHD?’ (Sleath et al., 2017) Health literacy has been proposed as an important mediator of child healthcare outcomes and it is clear there is further work to be done in providing better psychoeducation about ADHD (Sanders et al., 2009). Greater understanding of ADHD as a potential long‐term condition should inform young people, carers and clinicians' decisions about continuing medication (Price, Newlove‐Delgado, et al., 2019).

Clinicians in children's services should periodically revisit young people's understanding of ADHD at different developmental stages. Similarly, medication breaks during weekends or holidays may reinforce the misconception that medication is only necessary to cope with school. Clinicians should consider trials without medication (including during the school day as appropriate) prior to the crucial transition period (Brinkman et al., 2012; Conrad, 1985). The process of transition should start in the early teens, but ideally be completed by the age of 18(Yassaee et al., 2019); whilst many young people will experience fewer symptoms, a significant group will benefit from continued use of medication, which should be formally assessed.

### 4.3 Life circumstance of the young person

The impact of changing life circumstances was a commonly occurring theme in all three groups. These changes often led to an involuntary termination of their ADHD medication which subsequently had a detrimental impact on these young people's lives. Professionals need to have a greater awareness of the impact of the life events and life choices that young people make on whether they continue or stop ADHD medication. Discussion of education, occupation, pregnancy, relationships, driving and criminal justice involvement should all be central to the assessment and transition of young people with ADHD.

### 4.4 Challenges young people faced in accessing services

The CATCh‐uS study was developed to identify the challenges in young people's transition between children's and adult ADHD services (Janssens et al., n.d.). The challenges faced by young people returning to services is in keeping with findings from qualitative research into adult ADHD services (Matheson et al., 2013; Price, Janssens, et al., 2019). In many cases, guidelines for optimal transition were not followed, which resulted in loss to follow up (National Institute for Health and Care Excellence (NICE), 2016, 2018). Difficulties with access to services, or returning to services, not only impede access to ADHD medication but may also increase disengagement outside of transition. For example, the cumulative costs of prescriptions, or the time taken to travel to appointments on a regular basis may become overwhelming. The core impairments of ADHD mean that although chronologically adult, these young people will find it harder to organize themselves and deal with complexity than their peers. Clarity regarding transition processes in local areas and better preparation of young people and their parents/carers for the process of transition, including advice on what to expect from adult services, are essential.

### 4.5 Relevance to other long‐term conditions

The perceived balance between benefits and adverse effects of medication, changes in life circumstances and challenges in accessing services all may apply to young people on long‐term medication for other chronic health conditions. For example, children with epilepsy who viewed their medications as a cure were likely to stop medication once their symptoms were controlled; but over time many children with epilepsy develop the understanding that medicine is a preventative measure rather than a cure (Webster, 2017).

### 4.6 Limitations of the study

Our research benefitted from a robust analysis of a large qualitative purposive sample designed to reflect the breadth of experiences among young people taking ADHD medication in the UK. However, the study only interviewed young people that were engaged with services, and excluded young people who remained out of services, who may have different experiences of medication discontinuation, or ADHD (see Figure 1). Due to the design of the study we were unable to separate data on stimulant and non‐stimulant medication, and our findings may not reflect the experiences of young people with ADHD in other contexts.

### 4.7 Future research directions

This study demonstrates the importance of appropriate psychoeducation around ADHD for young people and their families. Further research into the views and experiences of front‐line clinicians working with young people with ADHD would help to ensure that these clinicians share an understanding of the importance of continuing ADHD medication into adulthood where appropriate and to understand and develop service models that supported their involvement.

Our finding that life events are a common reason for young people to stop medication for ADHD has not previously been reported. This study has highlighted that particular groups are likely to be vulnerable to discontinuation at particular times, for example, students with split residency between university and the parental home, those leaving care, or those in prison or homeless. Further research into these vulnerable groups would inform practice to better support these individuals.

One approach to support the continuation of ADHD medication in later adolescence is the development of services for young people that continue to the age of 25. This may help to reduce the drop out from services in the mid‐to‐late teens and early twenties, especially if these services are developed with the access needs of young people in mind. The use of ADHD medication in alternative service models needs evaluation, and it is important that the barrier of transition is not merely kicked further down the age range.

## FUNDING INFORMATION

This study/project is funded by the National Institute for Health Research (NIHR) Health Services and Delivery Research (HS&DR) CATCh‐uS, REF: 14/21/52. The views expressed are those of the author(s) and not necessarily those of the NIHR or the Department of Health and Social Care.

## CONFLICT OF INTEREST

Tamsin Ford received an honorarium from Takeda to talk about transition in ADHD at their Nurses Forum 1 March 2020. These funders had no influence on the CATCh‐uS study results or the analysis presented in this paper.

## ETHICS STATEMENT

The authors assert that all procedures contributing to this work comply with the ethical standards of the relevant national and institutional committees on human experimentation and with the Helsinki Declaration of 1975, as revised in 2008. All procedures involving human subjects/patients were approved by the NRES South Yorkshire Ethics Committee: Yorkshire & The Humber (REC Reference: 15/YH/0426) and the University of Exeter Medical School Research Ethics Committee (REC Application Number: 15/07/070). Written informed consent was obtained from all participants prior to the interview taking place. Parents of young people aged 14 to 16 years old consented for their child to take part; researchers sought assent from the young person. A £10 shopping voucher was offered to thank young people and support their recruitment.

## AUTHORS' CONTRIBUTIONS

TF and AJ designed and led the original CATCh‐uS study. DT, JG and AJ formulated the research question for the secondary analysis. HE, AP and AJ conducted semi‐structured interviews and completed primary analysis of the data. DT and JG completed a secondary analysis of the data for this paper. All authors were involved in the writing and editing of the final manuscript. All authors gave approval to the submitted manuscript.

## Data Availability

The qualitative data on which this analysis is based is archived at the University of Exeter Medical School. Given the detailed nature of the transcripts, these data are not publically available but permission to access can be sought via catchus@exeter.ac.uk.
